# Microbes Bind Complement Inhibitor Factor H *via* a Common Site

**DOI:** 10.1371/journal.ppat.1003308

**Published:** 2013-04-18

**Authors:** T. Meri, H. Amdahl, M. J. Lehtinen, S. Hyvärinen, J. V. McDowell, A. Bhattacharjee, S. Meri, R. Marconi, A. Goldman, T. S. Jokiranta

**Affiliations:** 1 Haartman Institute, Department of Bacteriology and Immunology and Immunobiology Research Program, University of Helsinki, Helsinki, Finland; 2 Institute of Biotechnology, University of Helsinki, Helsinki, Finland; 3 Virginia Commonwealth University Medical Center, Richmond, Virginia, United States of America; 4 Department of Biosciences, Division of Biochemistry and Biotechnology, University of Helsinki, Helsinki, Finland; National Institute of Allergy and Infectious Diseases, National Institutes of Health, United States of America

## Abstract

To cause infections microbes need to evade host defense systems, one of these being the evolutionarily old and important arm of innate immunity, the alternative pathway of complement. It can attack all kinds of targets and is tightly controlled in plasma and on host cells by plasma complement regulator factor H (FH). FH binds simultaneously to host cell surface structures such as heparin or glycosaminoglycans *via* domain 20 and to the main complement opsonin C3b *via* domain 19. Many pathogenic microbes protect themselves from complement by recruiting host FH. We analyzed how and why different microbes bind FH via domains 19–20 (FH19-20). We used a selection of FH19-20 point mutants to reveal the binding sites of several microbial proteins and whole microbes (*Haemophilus influenzae, Bordetella pertussis*, *Pseudomonas aeruginosa*, *Streptococcus pneumonia*, *Candida albicans*, *Borrelia burgdorferi*, and *Borrelia hermsii*). We show that all studied microbes use the same binding region located on one side of domain 20. Binding of FH to the microbial proteins was inhibited with heparin showing that the common microbial binding site overlaps with the heparin site needed for efficient binding of FH to host cells. Surprisingly, the microbial proteins enhanced binding of FH19-20 to C3b and down-regulation of complement activation. We show that this is caused by formation of a tripartite complex between the microbial protein, FH, and C3b. In this study we reveal that seven microbes representing different phyla utilize a common binding site on the domain 20 of FH for complement evasion. Binding *via* this site not only mimics the glycosaminoglycans of the host cells, but also enhances function of FH on the microbial surfaces *via* the novel mechanism of tripartite complex formation. This is a unique example of convergent evolution resulting in enhanced immune evasion of important pathogens *via* utilization of a “superevasion site.”

## Author Summary

Complement is an important arm of innate immunity. Activation of this plasma protein cascade leads to opsonization of targets for phagocytosis, direct lysis of Gram-negative bacteria, and enhancement of the inflammatory and acquired immune responses. No specific signal is needed for activation of the alternative pathway of complement, leading to its activation on all unprotected surfaces. Pathogenic microbes need to evade this pathway, and several species are known to recruit host complement inhibitor factor H (FH) to prevent the activation. FH is important for protection of host cells, too, as defects in FH lead to a severe autoreactive disease, atypical hemolytic uremic syndrome. We have now identified at the molecular level a common mechanism by which seven different microbes, *Haemophilus influenzae, Bordetella pertussis, Pseudomonas aeruginosa, Streptococcus pneumoniae*, *Candida albicans, Borrelia burgdorferi* and *B. hermsii*, recruit FH. All microbes bind FH *via* a common site on domain 20, which facilitates formation of a tripartite complex between the microbial protein, the main complement opsonin C3b, and FH. We show that, by utilizing the common microbial binding site on FH20, microbes can inhibit complement more efficiently. This detailed knowledge on mechanism of complement evasion can be used in developing novel antimicrobial chemotherapy.

## Introduction

Complement system (C) is an important part of innate immunity in human plasma, and the alternative pathway of complement (AP) is the first line of defense against invading microbes. AP is spontaneously activated on all unprotected surfaces leading to covalent binding of the main complement opsonin C3b to hydroxyl or amine groups. Surface-attached C3b forms a base for enzymatic convertases, which cleave intact C3-molecules until the activator surface is covered with C3b-molecules. This opsonization leads to opsonophagocytosis, propagation of the cascade resulting in release of chemotactic and anaphylatoxic peptides, and formation of lytic membrane attack complexes. To prevent attack against host structures and over consumption of the components in plasma, complement needs to be tightly regulated.

The main regulator of the AP in plasma is factor H (FH). FH is a 150 kDa glycoprotein and consists of twenty globular complement control protein modules (CCPs), each approximately 60 residues long. The AP control activity of FH is in domains 1–4 (FH1-4) [1], [2]. The so-called cofactor activity of FH is needed for inactivation of the central complement opsonin C3b by the serine-protease factor I. In addition to this, FH regulates AP activation by competing with factor B in binding to C3b and accelerating the decay of AP convertase C3bBb [3], [4]. To regulate complement, FH has to discriminate between host and non-host surfaces, as activation is warranted on microbial surfaces, but obviously not on host surfaces. This “target recognition” site is known to be in the carboxyl-terminal domains 19–20 (FH19-20) [5], [6]. Our structures of domains 19–20 alone [7] and complexed with C3d [8] showed how SCR20 can bind to cellular and glycosaminoglycan containing surfaces while SCR19 binds simultaneously to C3d part of C3b facilitating control of the AP. This dual binding ability facilitates target recognition by the AP.

The necessity of FH and its ability to distinguish between host and non-host surfaces is demonstrated by mutations in the carboxyl-terminus of FH. Even heterozygous mutations in this region can lead to uncontrolled AP activation on host cells causing severe damage to endothelial cells, red cells, and platelets, resulting in a serious systemic disease, atypical hemolytic uremic syndrome [9]. Another important target binding region in FH is within domain 7 and polymorphism in this domain is strongly associated with age-related macular degeneration, the most common cause of blindness in elderly people in industrialized countries [10], [11].

FH is utilized by several pathogenic microbes for protection against complement attack [12]. Binding of FH down regulates opsonization and prevents further amplification of the C cascade followed by formation of cytolytic membrane attack complexes. While prevention of opsonization and subsequent phagocytosis is beneficial for practically all microbes, evasion of membrane attack complex formation is especially important for Gram-negative bacteria and spirochetes. Acquisition of FH is important or even essential for pathogens; increasing numbers of them have been shown to bind FH [12]. There are two main interaction sites on FH for microbial binding (Table S1); one is within domains 6–7, and group A streptococci [13] and *Neisseria* [14], for example, utilize this site. Binding via domains 6–7 facilitates also utilization of FHL-1, an alternatively spliced transcript derived from FH-gene which contains domains 1–7 of FH and has cofactor-activity like FH [15]. Many microbes have been shown to bind both FH and FHL-1 [16].

The other microbial interaction site on FH is in the carboxyl-terminal domains 19–20. It seems that most microbes utilize both sites: for instance, *B. burgdorferi* sensu stricto, which causes Lyme disease, binds FH *via* domain 7 using protein CRASP-1 [17] and via domains 19–20 using outer surface protein E (OspE) and its paralogs [18]. This ability for dual binding facilitates efficient protection against the AP attack. Due to the high homology between the C-terminus of FH and C-termini of FH-related proteins (FHRs), some microbes bind also certain FHRs but the significance of this phenomenon for immune evasion is not clear yet.

We wanted to analyze in detail how and especially why different microbes utilize FH *via* the carboxyl-terminus. We selected pathogens representing Gram-negative, Gram-positive, and eukaryote microbes known to bind FH, and three microbial proteins, OspE (from *B. burgdorferi* sensu stricto) [18], FhbA (from *B. hermsii*) [19], and Tuf (from *P. aeruginosa*) [20]. We discovered that they all share a common binding site in domain 20 that overlaps but is not identical with the heparin and cellular binding sites. We also showed that FH bound to the microbial binding site forms a tripartite complex with C3b and furthermore, formation of this complex not only facilitates regulation of the AP but also enhances it.

## Results

### A common microbial binding site on FH domain 20

We first characterized at the molecular level how microbes bind FH *via* domains 19–20. We generated point mutations to 14 surface exposed residues of a recombinant fragment of FH domains 19–20 and used five different microbes isolated from patients: three Gram-negative bacteria *P.aeruginosa* (*Pa*) [20], (*H. influenzae* (*Hi*) [21], *B. pertussis* (*Bp*) [22]), one Gram-positive bacterium (*S. pneumoniae* (*Sp*) [23]), and one eukaryotic pathogen (*C. albicans* (*Ca*) [24]). We also measured binding of full FH to strains used and noticed they all bind FH, as expected on the basis of previous reports (Figure S1).

**Figure 1:**
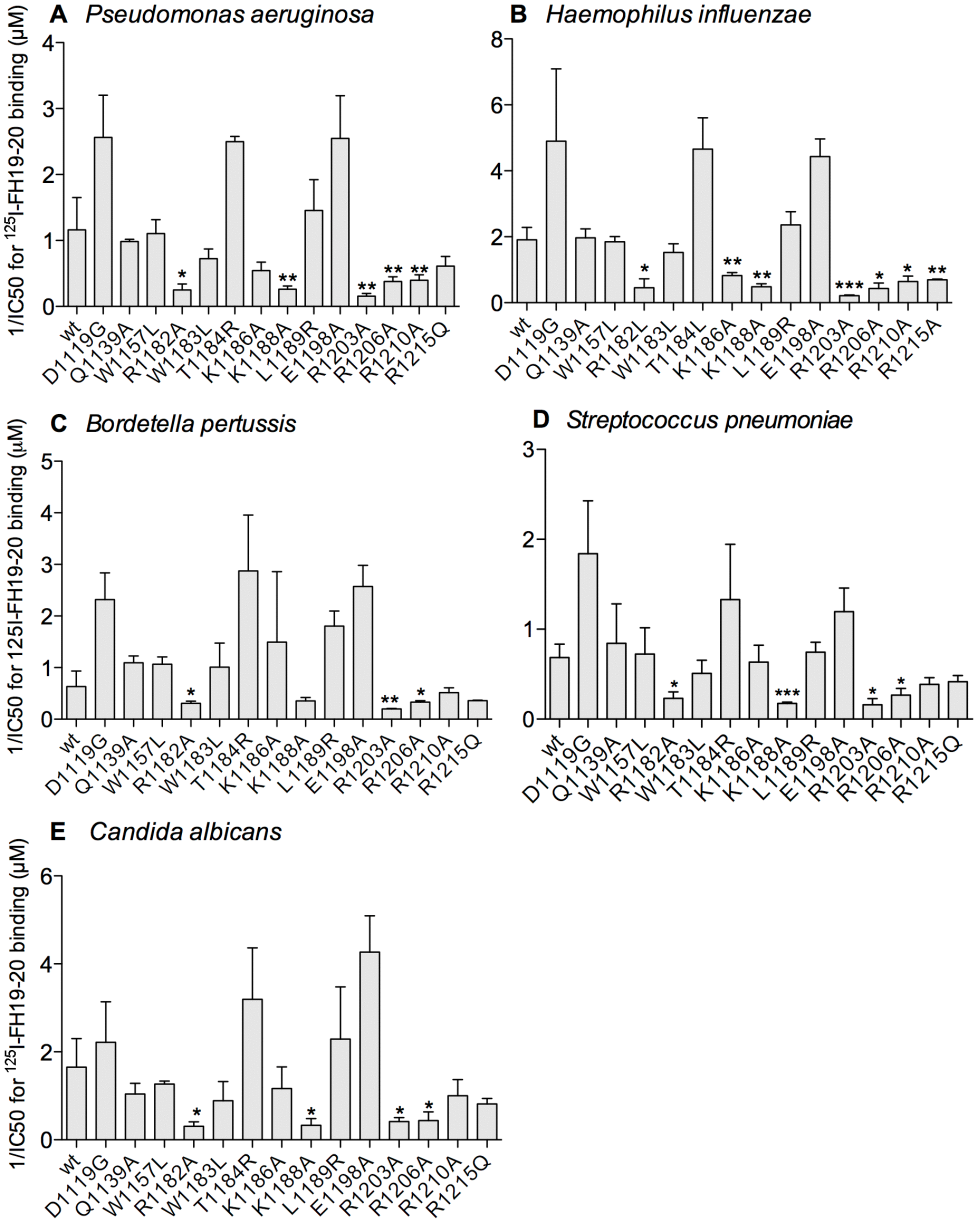
Microbial binding site on FH19-20. *Pseudomonas aeruginosa* (**A**), *Haemophilus influenzae* (**B**), *Bordetella pertussis* (**C**), *Streptococcus pneumoniae* (**D**), and *Candida albicans* (**E**) were coated to microtitre plates and binding of ^125^I-FH19-20 was measured in the presence of serial dilutions of 14 mutant proteins. Bound radioactivity was measured and the IC50 values (the 50% inhibitory concentration in µM) were determined by fitting these measurements to inhibition curves (Figure S2). Means of the reciprocal values of IC50 (1/IC50) with SDs of three individual experiments performed in triplicate are shown with difference compared to the wildtype (wt) calculated by a t-test. *p<0.05, **p<0.01, ***p<0.001.

Binding of ^125^I-labeled wild type (wt) FH19-20 was measured in the presence of increasing amounts (up to 7 µM) of the mutant FH19-20 constructs. Concentrations of the mutants that inhibited 50% of the wt FH19-20 binding (IC50) were calculated from binding curves of three experiments done in triplicate (examples are shown in Figure S2) and shown in Figure 1 as a reciprocal value (1/IC50) for clarity (diminished value indicating diminished binding). Three mutations, R1182A, R1203A, and R1206A, caused decreased binding to all five microbes (p<0.05); K1188A had reduced binding to four microbes (*Hi, Pa, Sp, Ca*); R1210A to three (*Hi, Pa, Sp*); and the K1186A and R1215Q mutations reduced binding to one microbe (*Hi*) (Figure 1). Four other mutations (W1183L, T1184R, L1189R, E1198A) in domain 20 and three (D1119G, Q1139A, W1157L) in domain 19 showed no reduction in binding compared to wt.

**Figure 2:**
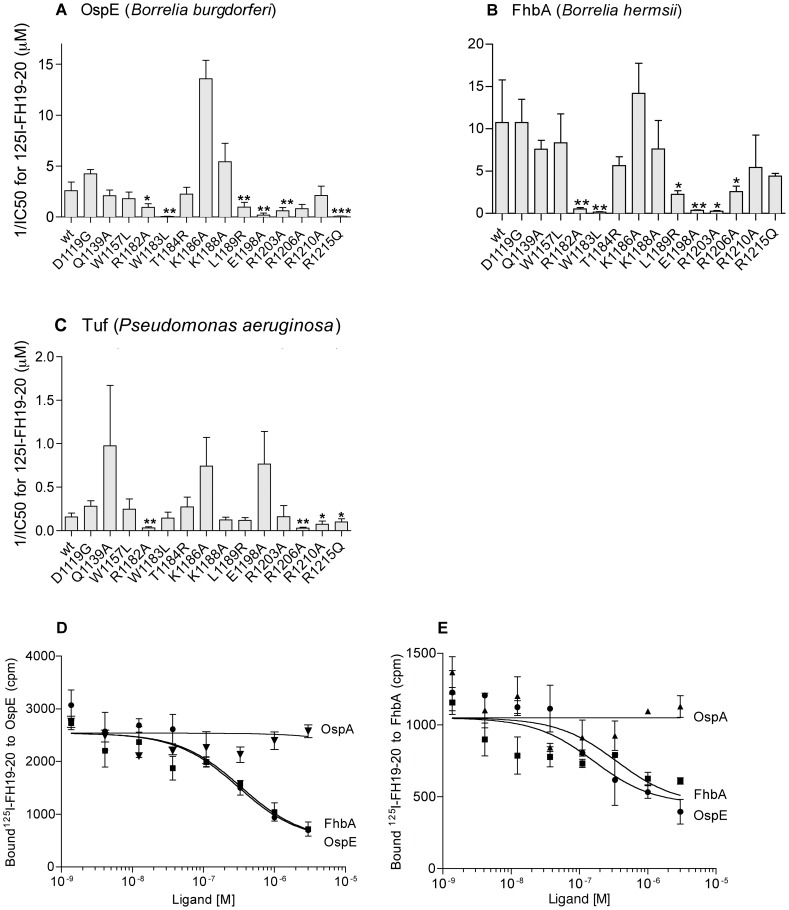
Binding site for microbial proteins on FH19-20. Three recombinant FH19-20 binding microbial proteins, OspE (**A, D**), FhbA (**B, E**) and Tuf (**C**) were coated to microtitre plates. First, inhibition of binding of ^125^I-FH19-20 by the FH19-20 mutants (**A–C**) was measured, data fitted to inhibition curves and IC50 values (the 50% inhibitory concentration) determined. Data shown are means of 1/IC50 values from three experiments performed in triplicate (bars indicating SDs); differences compared to wt were calculated by a t-test (*p<0.05, **p<0.01, ***p<0.001). Second, inhibition of binding of ^125^I-FH19-20 to OspE (**D**) or FhbA (**E**) by microbial proteins, OspE, FhbA, and a negative control OspA, was measured. Data from representative experiments performed in triplicate are shown with SDs (**D, E**).

To further characterize interaction of FH with microbial surfaces, similar binding inhibition assays were used with three non-homologous and structurally unrelated bacterial outer surface proteins: OspE, a 15 kDa protein from a Lyme borreliosis agent *B. burgdorferi* [18], FhbA, a 20 kDa protein from a relapsing fever spirochete *B. hermsii* [25], and Tuf, a 43 kDa protein from *P. aeruginosa* [20]. Binding of ^125^I-FH19-20 to the recombinant proteins was measured in the presence of increasing concentrations of the 14 mutant proteins and the IC50 values were calculated from the binding curves as above. When compared to wt FH19-20, two mutant proteins, R1182A and R1206A, showed decreased affinity to all the three microbial proteins, five mutants (W1183L, L1189R, E1198A, R1203A, R1215Q) to two microbial proteins and one mutant (R1210A) to one protein (p<0.05) (Figure 2, Panels A–C, shown as a reciprocal value (1/IC50) for clarity). The effect of three mutants (T1184R, K1186A, K1188A) in domain 20 and three (D1119G, Q1139A, W1157L) in domain 19 was comparable to wt FH19-20 (p>0.05). Six of the mutants showed decreased binding to both OspE and FhbA suggesting an overlap of the binding sites. The overlap was confirmed using cross inhibition assays with OspE and FhbA (Figure 2, Panels D and E).

**Figure 3:**
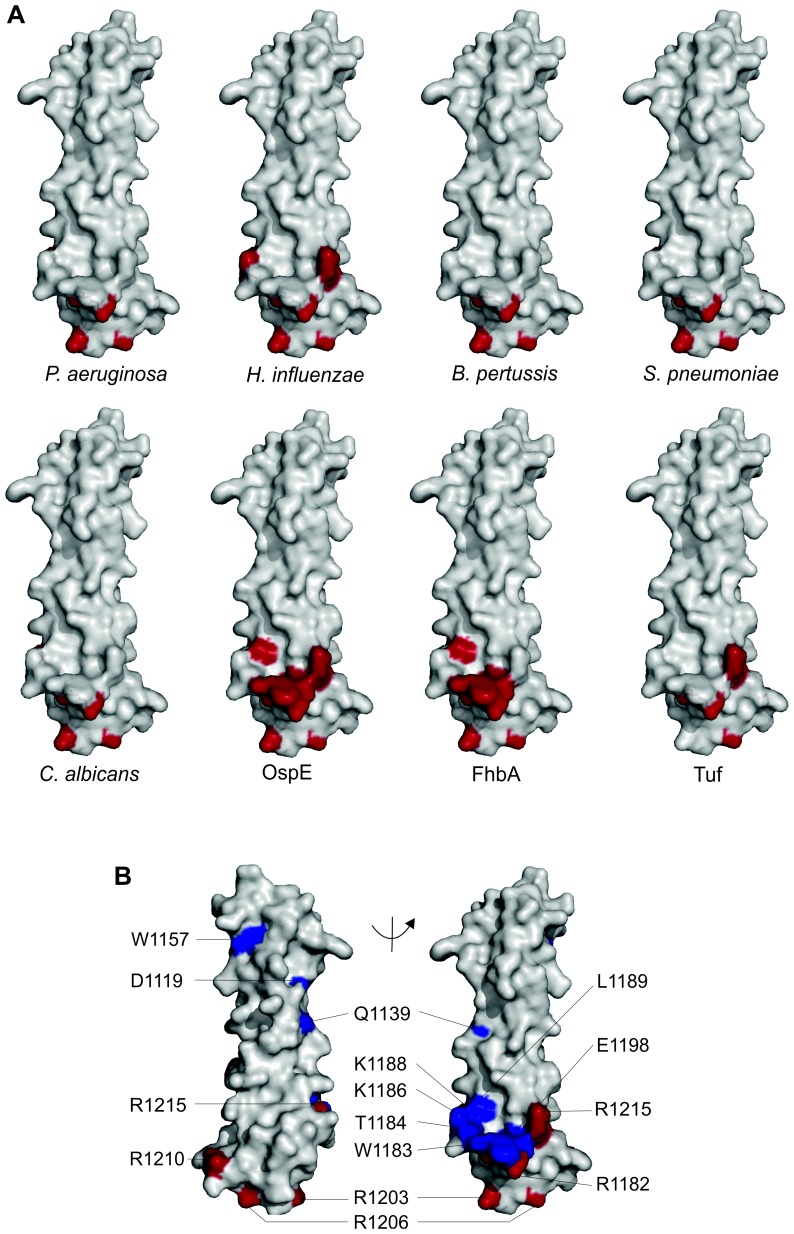
Microbial binding site on the structure of FH20. Panel **A** shows location of the binding sites of *Pseudomonas aeruginosa*, *Haemophilus influenza, Bordetella pertussis, Streptococcus pneumoniae, Candida albicans*, OspE, FhbA, and Tuf on the surface of the crystal structure of FH19-20 [7]. The involved residues are shown in red and in each figure the FH domain 19 is on the top and domain 20 on the bottom. In the panel **B** the common microbial binding site is marked on the surface model of FH19-20. Residues affecting binding of FH19-20 to three or more microbes (Table 1) are marked in red, other analyzed mutated residues are marked in blue and all residues have been annotated.

Taken together, the binding inhibition assays revealed that all mutants that affected binding were in the domain 20. Furthermore, we identified one mutant (R1182A) with significantly decreased binding to all the microbes or microbial proteins analyzed and two mutants (R1203A, R1206A) with significantly reduced binding to seven out of eight targets (p<0.05) (Table 1). In addition, the three central residues in microbial binding, R1182A, R1203A, and R1206A, are close to each other in the crystal structure of FH19-20 [7]. They are within 14 Å of each other on domain 20 and three residues (K1188A, R1210A, R1215Q) involved in binding to several microbes are also nearby (Figure 3). Folding of all these mutants was comparable to wt FH19-20 according to a circular dichroism analyses (Figure S3).

**Table 1:** Summary of the FH19-20 binding results.

FH19-20 mutation	Pa	Hi	Bp	Sp	Ca	OspE	FhbA	Tuf
D1119G	0.3*	0.4	0.3	0.4	0.7	0.6	0.9	0.5
Q1139A	0.8	1.0	1.1	0.9	1.4	1.2	1.1	0.2
W1157L	0.7	1.0	0.6	1.1	1.2	1.4	1.4	0.7
R1182A	**4.0**	**4.9**	**1.9**	**3.3**	**3.9**	**3.0**	**18.0**	**5.0**
W1183L	1.1	1.2	0.7	1.5	2.0	**97.1**	**66.4**	1.2
T1184R	0.3	0.4	0.2	0.6	0.5	1.2	1.7	0.6
K1186A	1.6	**2.3**	0.7	1.2	1.4	0.2	0.5	0.2
K1188A	**3.5**	**4.0**	1.7	**4.1**	**4.1**	0.8	1.6	1.3
L1189R	0.6	0.8	0.4	1.0	0.8	**2.9**	**4.9**	1.3
E1198A	0.3	0.4	0.3	0.6	0.4	**24.7**	**25.1**	0.2
R1203A	**6.2**	**8.6**	**3.2**	**5.0**	**3.5**	**4.7**	**41.9**	1.5
R1206A	**2.2**	**4.8**	**2.0**	**2.9**	**3.7**	**3.4**	**3.1**	**3.1**
R1210A	**2.1**	**3.0**	1.5	**2.0**	1.6	1.3	1.8	**2.3**
R1215Q	1.7	**2.5**	1.4	**1.7**	1.8	**33.8**	2.0	**1.6**

* The values represent relative binding of FH19-20 mutants *vs.* wild type FH19-20 (IC50mut/IC50wt) to microbes (Pa; *Pseudomonas aeruginosa*, Hi; *Haemophilus influenzae*, Bp; *Bordetella pertussis*, Sp; *Streptococcus pneumoniae*, Ca; *Candida albicans*) or microbial proteins (OspE, FhbA, Tuf). Bold font of the value indicates statistically significant increase in IC50 (i.e. diminished binding) when compared to wild type FH19-20 (unpaired t-test, p<0.05).

### Microbes mimic host surfaces upon binding to FH20

**Figure 4:**
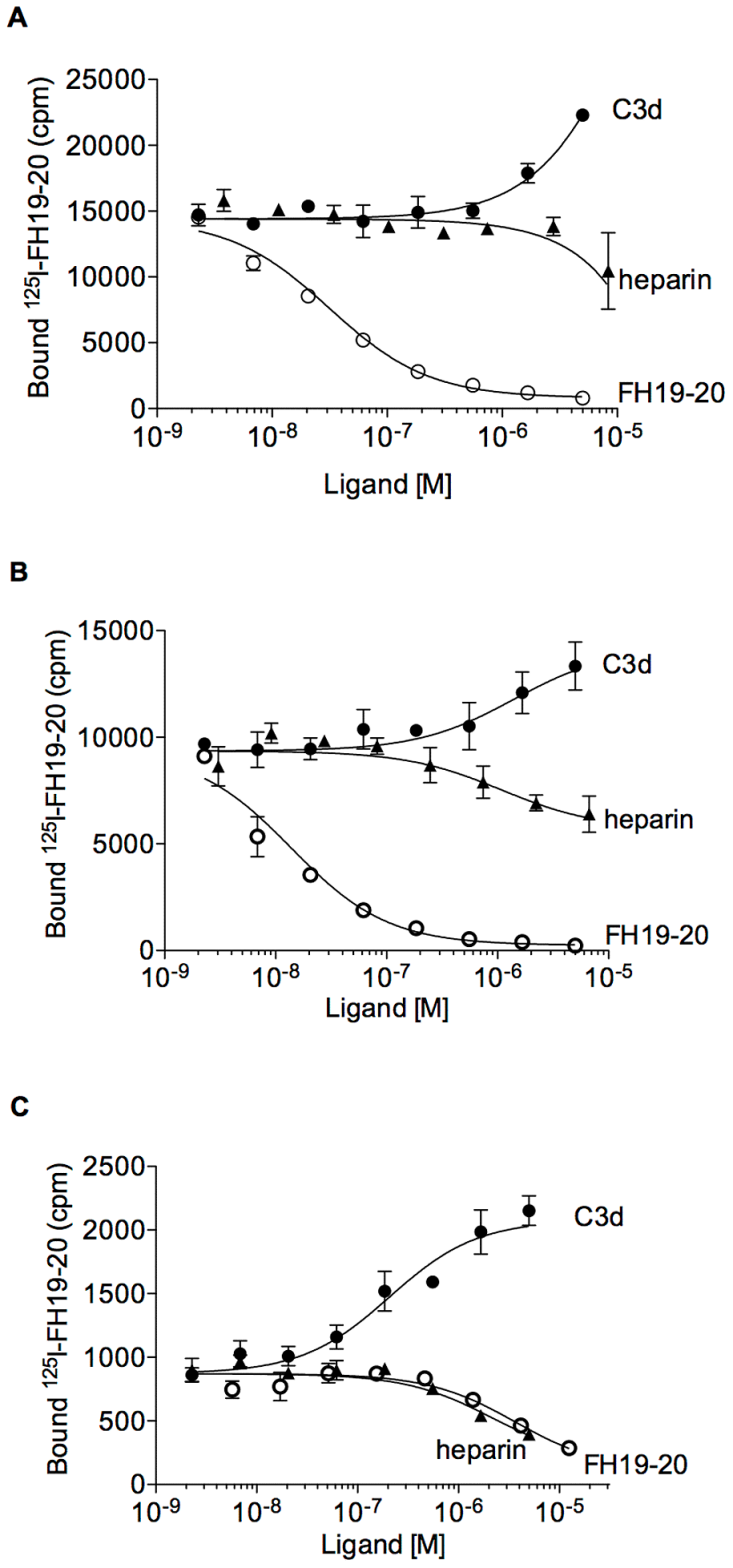
The common microbial binding site on FH20 overlaps partially with the heparin but not the C3d binding site. Effect of increasing concentrations of C3d, heparin, and FH19-20 in binding of ^125^I-FH19-20 to solid phase OspE (panel **A**), FhbA (panel **B**), or Tuf (panel **C**) is shown (counts per minute (cpm) ± SD from a representative of three experiments performed in triplicates is shown).

One binding site for glycosaminoglycans/heparin is located at FH domain 20 [26]. We next analyzed if microbes could utilize this site by analyzing binding of ^125^I-FH19-20 to OspE, FhbA, and Tuf in the presence of heparin, a model substance for cell surface glycosaminoglycans. We showed that heparin inhibits binding of FH19-20 efficiently to Tuf and slightly also to OspE and FhbA (Figure 4). The data are consistent with previous data showing that glycosaminoglycans bind to residues R1203, R1206, R1210, and R1215 at the very carboxyl-terminus of FH20 [27]. This suggests that the microbial binding site on FH overlaps to some extent with, but is not identical to, the heparin binding site needed for recruitment of FH to eliminate C3b on host cells.

Down-regulation of the AP by FH on host cells occurs because FH20 binds to glycosaminoglycans/heparin while FH19 binds simultaneously to the C3d part of C3b [8], [28]. Next we tested if microbes could utilize FH similarly, i.e. facilitating a two point binding of FH19-20 to surface-bound C3b, one site binding to the microbial protein and the other to C3b. There are two binding sites on FH19-20 for the central complement opsonin C3b, one in domain 19 and the other in domain 20 [8]. Structural analysis shows that the site on domain 20 overlaps with the microbial site, while the site on domain 19 of FH is clearly distinct from it [8]. In agreement with our model, binding of C3d did not inhibit binding of FH19-20 to the microbial proteins (Figure 4, panels A–C) but, to our surprise, actually enhanced it.

### Formation of a tripartite complex between microbial protein, FH19-20, and C3b

**Figure 5:**
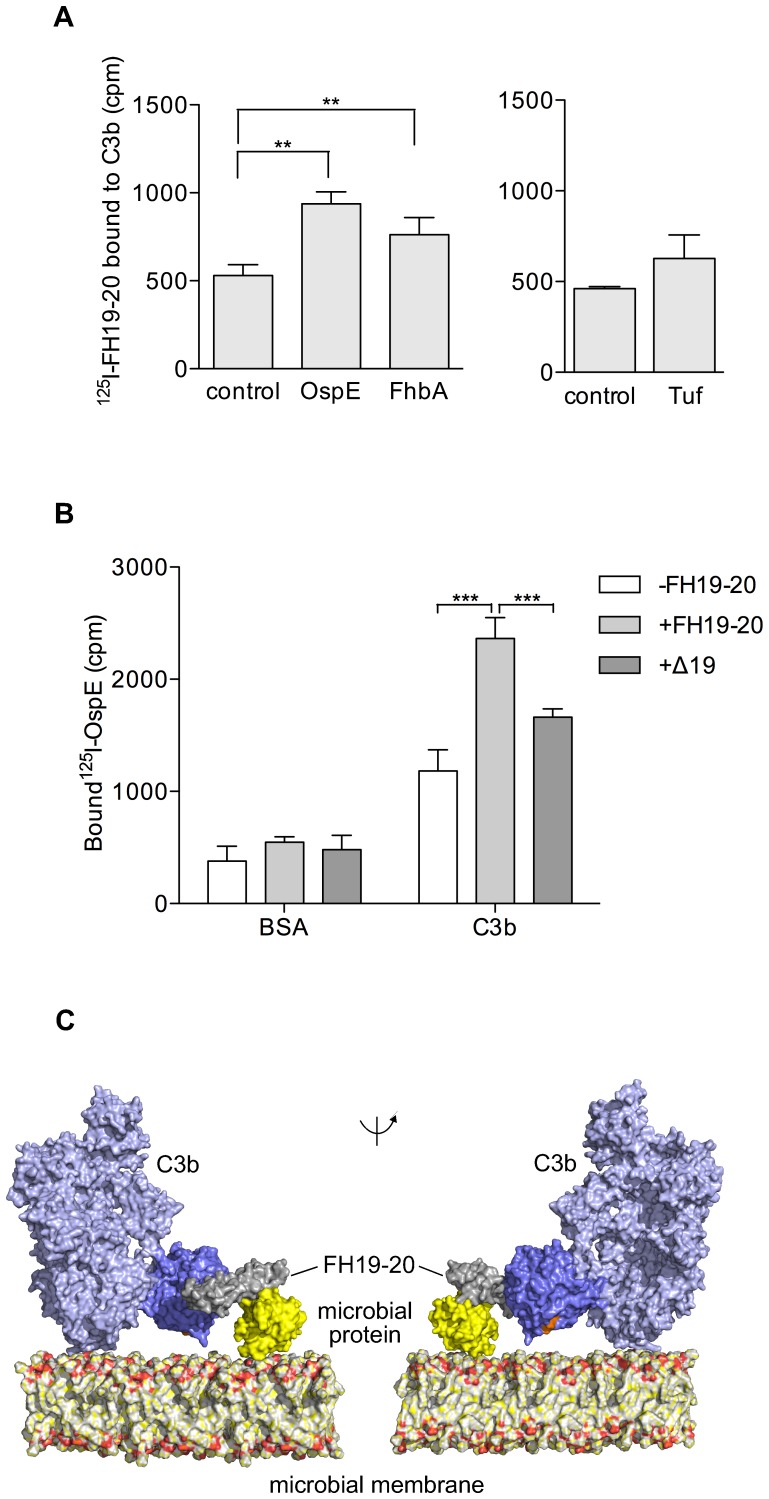
Binding of FH20 to microbial proteins enhance the FH-C3b interaction. Panel **A** shows enhanced binding of radiolabeled FH19-20 to solid phase C3b in the presence of 1.25 µM OspE, FhbA, or Tuf compared to buffer control (cpm ±SD from a representative experiment performed in triplicates is shown; difference to the control was calculated by a t-test; *p<0.05, **p<0.01, ***p<0.001). In panel **B**, binding of ^125^I-OspE to C3b (or bovine serum albumin, BSA, as a negative control) is shown in the presence or absence 1.25 µM of FH19-20 or FH19^Del^-20 lacking the C3d binding site on FH domain 19 (cpm ±SD from a representative experiment performed in triplicates is shown). Panel C shows solvent accessible surface representation of a model of the tripartite complex between FH19-20, C3b, and a microbial protein on a microbial surface. Two projections with the microbial membrane lipid bilayer on the bottom are shown. Color code: C3b (2WII, [44]) is shown in blue and its C3d part (TED domain) is darker blue with the thioester site in orange (1C3d, [7]); a microbial protein is shown in yellow; FH19-20 is shown in grey (2g7i, [8]).

As C3d enhanced binding of FH19-20 to microbial proteins, we analyzed further if microbial proteins could enhance binding of FH19 to its main physiological ligand, C3b. We measured the binding of ^125^I-FH19-20 to C3b in the presence of OspE, FhbA, and Tuf. OspE and FhbA enhanced binding of FH19-20 to C3b statistically significantly while enhancement with Tuf was smaller and not significant (Figure 5, Panel A). This suggests that a microbial protein, FH19-20, and C3b together form a tripartite complex. We were able to prove this by measuring binding of ^125^I-OspE to solid phase C3b in the presence of FH19-20 (Figure 5, Panel B). This means that the tripartite complex must form, because OspE alone does not bind C3b [18]. Mutation of four central residues in the C3d/C3b binding site on domain 19 of FH (FH19^del^-20) [8] significantly reduced the formation of the tripartite complex, indicating that the C3d/C3b binding site on domain 19 is essential for the interaction (Figure 5, Panel B). These experiments show that FH19-20 can bind simultaneously to a microbial protein and C3b, and that binding of microbial proteins to FH19-20 enhances the FH-C3b interaction. To further test formation of the tripartite complexes on microbial surfaces we measured effect of C3d (100 µg/ml) on binding of FH19-20 to the surface of whole microbes (*B. burgdorferi, S. pneumoniae, P. aeruginosa, H. influenzae* and *C. albicans*). A small increase in FH19-20 binding was observed with all the used microbes, most clearly with *S. pneumoniae* and *C. albicans* (Figure S4). No binding of ^125^I-C3d to any microbes was seen (data not shown).

By modeling the tripartite complex on a surface using the structure of FH19-20 in complex with C3d [8], C3b (containing the C3d part) [29], and our recent crystal structure of FH19-20 in complex with borrelial OspE protein (Bhattacharjee et al., submitted), a model of a microbial surface protein, we could also show that formation of a tripartite complex is possible without any steric clashes. Furthermore, in this model the thioester site of C3b faces towards the membrane indicating that a surface-bound microbial protein can enhance binding of FH to C3b on the same surface (Figure 5, Panel C).

### Binding to a microbial protein enhances the regulatory function of FH

**Figure 6:**
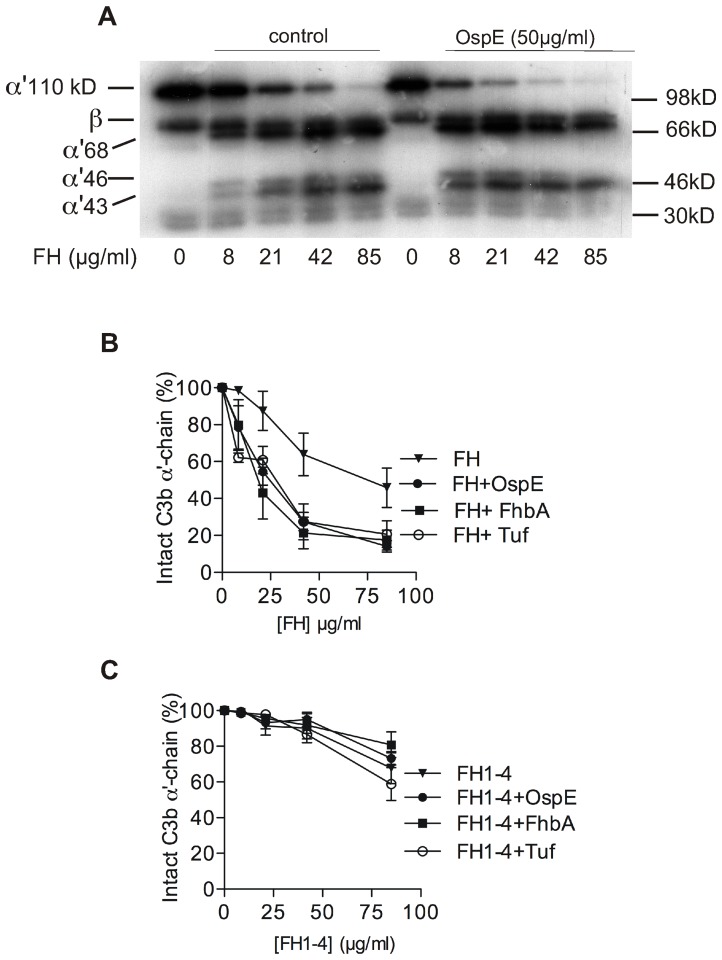
Enhanced cofactor-activity of FH bound to microbial proteins. Effect of OspE, FhbA, and Tuf (each 50 µg/ml) in elimination of C3b by FH (8–85 µg/ml) and factor I (15 µg/ml). Cleavage of the α′-chain of ^125^I-C3b was measured by evaluating the intensity of the α′-chain in autoradiography (example gel from one out of three experiments shown in panel **A** with mobility of the C3b fragments and size markers indicated) and intensity in the absence of FH was set as 100% (panel **B**). As a control, FH was replaced with recombinant FH1-4 fragment (**C**). Data in panels B and C are from three independent experiments with SDs indicated. * p<0.05, **p<0.01, ***p<0.001.

The results above suggested that, by enhancing the interaction between FH and C3b, microbes might be able to down-regulate complement activation more efficiently. The main regulatory function of FH is to act as a cofactor for serine protease factor I in inactivation of C3b. We therefore measured the cofactor activity of full length FH in factor I mediated cleavage of C3b in the presence of the three microbial proteins, OspE, FhbA, or Tuf (Figure 6, Panels A and B). All tested microbial proteins enhanced significantly the cofactor activity of FH (*p*<0.05 at ≥20 µg/ml for all of the proteins). The enhancement was due to the carboxyl-terminal part of FH, since it did not clearly occur when FH1-4 was used instead of full length FH (Figure 6, Panel C), *i.e.* enhancement obviously requires domains 19 and 20 that mediate formation of the tripartite complex.

## Discussion

Escape of the complement system, and especially its alternative pathway amplification cascade, is a prerequisite for microbial virulence since this first line immune mechanism is spontaneously activated on all non-protected surfaces. Microbes are known to protect themselves by binding host complement regulators from plasma or other body fluids: FH for protection against the alternative pathway activation and C4b-binding protein for inhibition of the classical and lectin pathways. Binding of FH has been thought to be simple recruitment of host FH onto the microbial surface since FH acts as a cofactor for factor I in the degradation of the central complement component C3b [30]. This inactivation is essential for microbial survival in nonimmune plasma or blood, since it prevents opsonophagocytosis and microbial lysis by the membrane attack complexes [31]. Microbes recruit host FH by binding it *via* two separate sites, one within the domains 6–7 and the other in the C-terminal FH19-20 (Table S1), but the reason for using these sites has remained unexplained.

Our new data show, first, that the microbes we studied not only use FH19-20, but in particular the same area on FH domain 20, which we have named the “common microbial binding site” (Figure 3, panel B). Second, our data show that binding *via* this particular site allows the formation of a tripartite microbial protein∶FH∶C3b complex (Figure 5, panel C). Third, and most importantly, our data show that formation of the tripartite complex enhances FH-mediated inactivation of C3b. This explains why many kinds of microbes have evolved to utilize this common microbial binding site on FH.

We analyzed the interaction site between the carboxyl-terminus of FH and microbes by measuring the effect of mutant FH19-20 proteins on binding of wt FH19-20 to five important human pathogens (Gram-negative and Gram-positive bacteria and a yeast). Next we analyzed FH19-20 binding by three structurally non-related, FH binding proteins, two from spirochetes, OspE from *B. burgdorferi* sensu stricto [18] and FhbA from *B. hermsii* [32], and Tuf from *P. aeruginosa* [21]. To our great surprise all the microbes and microbial proteins studied bound FH *via* heavily overlapping binding sites on domain 20 (Table 1, Figure 3, panel A). We found three key amino acids (R1182, R1203, R1206) that affected binding to *all* the studied microbes and three more (K1188A, R1210A, R1215A) that affected binding to at least three out of seven microbes analyzed. We believe that this site, the common microbial binding site, will be found to be used by many other pathogenic microbes too. We did not use full length FH with point mutations in these experiments since microbes have often two binding sites for FH (Table 1) and expression and purification of full-length FH with mutations in both the microbial binding sites might not result in easily interpretable results.

Since the different microbial proteins are non-homologous it is expected that they use slightly different residues within or next to the common microbial binding site on FH20 to form, for example, hydrogen bonds and hydrophobic contacts. An example of this is seen with OspE since mutations of two residues of FH19-20 (W1183 and E1198) that are not used by several other microbes had the most striking effect on OspE binding to FH19-20. Use of variable residues within the same area does not compromise the key finding that the used microbes share a common binding area on FH domain 20 but indicates variability in the structure of the microbial molecules binding to the common shared site on FH. It is obvious that only detailed structural analysis of different microbial FH-binding proteins in complex with FH19-20 will show how important each residue within or next to the common site is for the interaction.

At least three non-homologous microbial proteins and, in addition, four microbial species without known homologues of these proteins utilize the same site on FH20. For some of these microbes, it is not known which surface molecule recruits host FH and it is possible that, at least in some cases, the surface molecules are not proteins but carbohydrates. FH is known to bind to several negatively charged carbohydrates [33] and the common microbial binding site on FH20 overlaps with the site responsible for binding to at least one host carbohydrate, heparin (Figure 4, panels A–C) [27]. It remains to be studied if any microbe binds to the common microbial binding site on FH20 *via* a carbohydrate, and if carbohydrate binding to FH domain 20 could promote the FH∶C3b interaction through formation of a tripartite complex, similarly to the studied microbial proteins.

Why have different microbes evolved to utilize domain 20, and practically the same particular site on this domain, in recruitment of FH? Our results provide three reasons for this. First, our work shows that FH bound to microbial surface *via* domain 20 can also bind the C3d part of C3b by domain 19 (Figure 5, panels A and B). This brings FH near to its main target, C3b, and allows complement inhibition. On the basis of the superimposition of three structures, our recently solved structure of a microbial FH-binding protein (OspE) in complex with FH19-20 (Bhattacharjee et al, submitted) and the previously solved structures of FH19-20 in complex with C3d, [8] and of the C3b (containing the C3d [29]), it became clear that FH19-20 can bind simultaneously to a microbial protein and C3b (Figure 5, panel C). Furthermore, in this superimposition the microbial binding site is also directed towards the surface to which C3b is bound to *via* the thioester site and is therefore readily available for the microbial molecules in general. Second, the site on domain 20 is available under physiological conditions: the previously described physiologically important heparin binding site [27], [34], [35] and the common microbial site overlap to some extent (Figure 4, panels A–C). Ferreira and coworkers [36] have also emphasized that the cofactor site on domains 1–4 must not be disturbed upon binding of FH to a microbe and our common microbial binding site fulfills also this criterion. Third, utilization of this particular common microbial binding site provides more efficient down regulation of complement activation on the microbe. The intact α′-chain of C3b disappears more efficiently when FH and microbial proteins are present (Figure 6, Panels A and B). This is due to the carboxyl-terminus of FH, as practically no enhancement was seen by using FH1-4, which has cofactor activity but does not have the common microbial binding site on domain 20 (Figure 6, Panel C).

The tripartite microbial protein∶FH∶C3b/C3d complexes are clearly formed in fluid phase *in vitro* (Figure 5). It is, however, uneasy to demonstrate the complex formation on microbial surface since C3b is bound covalently to the molecules on the target surface and the formed tripartite complex is easily broken upon purification of C3b from the cells. To indicate the complex formation on cell surfaces we therefore used an experimental setup where binding of FH19-20 to whole intact microbes was analyzed in the presence of soluble C3d and saw that addition of C3d could, indeed, enhance the binding (Figure S4). Since the extrinsic C3d can enhance binding of FH onto the microbial surface it is highly likely that the microbial proteins could also enhance formation of the tripartite complex on the microbial surface, at least if the density of C3b depositions was high enough. The highest C3b concentration occurs on the target surface areas where alternative pathway activation is vigorously amplified via the feedback loop. It would be most beneficial for the microbe if the tripartite complexes were formed within those areas leading to maximal complement down regulation exactly at the spots where it is needed most. For the tripartite complex formation the microbial FH-binding molecule needs to be – or bend – next to the C3b molecule but most, if not all, of the FH-binding microbial proteins which have been structurally characterize are either long molecules (e.g. streptococcal M protein) or have a flexible tail that allows twisting and tilting (e.g. OspE, Bhattacharjee et al, submitted). Therefore at least some of the microbial FH-binding molecules seem to be able to operate on a broader area of the surface than just the exact spot they are attached to. The area where the tripartite complexes could be formed might in addition be expanded by lateral movement of the lipid tail or membrane anchor of the microbial FH-binding molecules on at least surface of Gram-negative bacilli.

An FH-related protein found in plasma, FHR-1, has a C-terminal domain that differs from domain 20 of FH only by two residues. The differences are located close to the microbial binding site and it remains to be studied if FHR-1 binds similarly to the used microbes, and if possible recruitment of FHR-1 is functionally beneficial or unfavourable for the microbes as FHR-1 does not have any cofactor-activity.

Clearly, formation of the tripartite complex is the reason for the increase in the regulatory function of FH caused by the microbial proteins. As far as we know, this kind of enhancement has neither been suggested, nor studied before. Instead, it has been suggested that microbes mimic host structures and thereby bind FH and other complement regulators [37]. Although microbes, heparin or endothelial cells do bind to overlapping sites on FH, this is not exactly molecular mimicry as the binding sites are not identical. The structures involved are completely different and they appear to differ from organism to organism. We and others have recently shown that host cells recruit FH *via* domain 20 [27], [35] and it remains to be studied if this leads to elevated FH function due to tripartite complex as in the microbial proteins [8], [28]. If this were the case, microbes utilizing the common microbial binding site on FH domain 20 would have *functional*, not molecular, mimicry of host cells. So far there is, however, no evidence of this.

The identified common microbial binding site on FH domain 20 represents a surprising type of host-pathogen contact – a single site on a host molecule utilized by several kinds of microbes in immune evasion. Such a common immune evasion site for both bacterial and eukaryotic pathogens has not been reported earlier. We call this kind of conserved site for microbial immune evasion a “superevasion site” and suggest that superevasion sites may occur on other powerful down regulators of host immunity, too. The concept of a microbial superevasion site is valid not only for down regulators of immunity, such as FH, but also for host immune activator molecules such as immunoglobulins. It is probable that, for example, staphylococcal protein A [38], streptococcal protein G [39], and *E. coli* protein EibD [40] are not the only microbial proteins that bind to a conserved site on IgG leading to prevention of the effector functions of immunoglobulins. This site on the Fc part of IgG is probably an example of a superevasion site on immune activator molecules.

In this study we have identified a conserved microbial binding site on domain 20 of the important complement regulator FH. We have shown that, by binding to the common binding site on FH, microbial proteins enhance the FH∶C3b interaction by enhancing their interaction, thereby increasing down regulation of C3b and leading to efficient evasion of complement attack and presumably to increased survival of the microbes in the host. The identified common microbial binding site on FH is the first example of a “superevasion site” pointing to new avenues not only in research on immune evasion by microbes but also in research aimed at novel vaccines and antimicrobial agents.

## Materials and Methods

### Proteins

The outer surface proteins OspE and OspA from *B. burgdorferi* sensu stricto strain N40 were cloned, expressed and purified as described [18]. FhbA was cloned and purified from *B. hermsii* strain MAN [32], and Tuf from a *P. aeruginosa* blood isolate strain similarly as described earlier [20]. Cloning and purification of wt FH19-20 and the FH19-20 mutants have been described earlier [7], [27], [41]. Circular dichroism spectras of six mutants (R1182A, W1183L, K1188A, E1198A, R1203A, R1206A) were compared to wt to confirm proper folding of the mutants (Figure S3, panel A). The capacity of these mutants to form oligomers was compared to wt FH19-20 using gel filtration on a Superdex 75 10/300 GL column (Figure S3, panel B). FH1-4 was produced as described [42]. C3 and FH were purified from human plasma and C3b generated with trypsin as described [43]. C3d was a kind gift from Prof. D. Isenman, Univ. of Toronto, Canada. Factor I was purchased from Calbiochem/MerckMillipore (Merck, Darmstadt, Germany) and BSA, gelatin and heparin from Sigma-Aldrich (St. Louis, MO, US). The wt FH19-20, FH, OspE, and C3b were labeled with ^125^I using the IodoGen method (Thermo Scientific Pierce, Rockford, IL, US).

### Bacteria

The strains of *Pseudomonas aeruginosa, Haemophilus influenzae, Streptococcus pneumoniae, Staphylococcus aureus* and *Candida albicans* we used were isolated from blood cultures of septic patients and were kind gifts of Dr. K. Haapasalo-Tuomainen, HUSLAB, Helsinki Univ. Central Hospital, and Univ. of Helsinki, Finland. *Bordetella pertussis* was a kind gift of Dr. Quishui He, Pertussis Reference Laboratory, Turku, Finland. The used serum sensitive *Haemophilus influenzae* strain is isolated from a throat swab of a healthy individual.

### Direct binding assays

To detect binding of FH or FH19-20 to the microbes, the bacteria and yeast were first washed three times with PBS. Approximately 1×10^8^ cells/reaction were incubated with radiolabeled FH or FH19-20 (40,000 cpm/reaction) in the absence or presence of C3d (0–100 µg/ml) in 50% PBS containing 0.1% gelatin (GPBS) at 37°C for 20 min with agitation (1,200 rpm). Cell-associated and free radioactive proteins were separated by centrifugation (10,000× *g*, 3 min) of the samples through 20% sucrose in GPBS. Radioactivities in the supernatant and pellet fractions were measured with a gammacounter (Wallac, Turku, Finland). The amounts of bound proteins were calculated as percentages of the total radioactivities in the corresponding pellets and supernatants. The experiments were performed three times in triplicate.

### Radioligand assays and data processing

Nunc Polysorp BreakApart plates (Thermo Scientific, Rockford, IL, US) were coated with either bacteria (1×10^6^/well in phosphate-buffered saline, PBS, at 37°C for 12 hours) or proteins (5–25 µg/ml in PBS at 4°C for 12 hours). The wells were blocked (0.5% BSA/PBS, 60 min at 22°C, or 0.5% BSA/50% PBS for the experiment shown in the Figure 4, Panel C) and washed with PBS. Serial dilutions of proteins were mixed with ^125^I-FH19-20 or ^125^I-OspE (50,000 cpm/well) in a separate 96-well microtitre plate (Greiner Bio One, Frickenhausen, Germany) before transferring into the coated wells. After incubation (37°C, 60 min) and washing with PBS (or 50% PBS for the experiment shown in Figure 4, Panel C), the radioactivity in each well was measured with a gamma-counter (Wallac, Turku, Finland). The inhibition curves were fitted using non-linear regression of a “log(inhibitor) vs. response” model using GraphPad Prism software (version 5.0b, GraphPad Software, CA, US). The mean inhibitory concentrations (IC50-values) were calculated from the fitted curves. All the assays were performed three times using triplicate wells.

### Cofactor assays

To measure cofactor activity ^125^I-C3b (100,000 cpm/assay) was mixed with factor I (16 µg/ml) in the absence or presence of FH or FH1-4 (8–85 µg/ml) and OspE, FhbA, and Tuf (50 µg/ml). Mixtures were incubated at 37°C for 5 min and, after adding β-mercaptoethanol, the samples were heated (3 min at 93°C) and run on 10% SDS-PAGE gels. The gels were subjected to autoradiography and cofactor activity was evaluated as the intensity of the C3b α′-chain measured with GelEval-programme (FrogDance Software, Dundee, UK).

### Statistical analyses

Values are expressed as means ± SD. All statistical analyses were performed using GraphPad Prism software and statistical differences were calculated with unpaired t-tests.

## Supporting Information

Figure S1 **Binding of full length FH to microbes used in the study.** Bacteria and yeast (1×10^8^/assay) were incubated with radiolabeled FH and samples were centrifuged through sucrose colums to separate unbound radioactivity. Amount of radioactivity in the pellet and supernatant was measured with a gamma-counter and FH bound to the microbes is shown as a percentage from total amount of protein given. Data (%) with SD's from a representative experiment performed in triplicates are shown. As negative controls a serum sensitive strain of *H. influenzae* and. *S. aureus* were used.
(PDF)

Figure S2 **Examples of the inhibition assays.** Curves from a single out of three experiments (performed in triplicates) where inhibition of ^125^FH19-20 binding to various microbes by wildtype (wt) and mutant FH19-20 proteins was analyzed to obtain IC50 values (shown in **Figure 1**). The used microbes were *Pseudomonas aeruginosa* (panels A and B), *Haemophilus influenzae* (panels C and D), *Bordetella pertussis* (panels E and F), *Streptococcus pneumoniae* (panels G and H), and *Candida albicans* (panels I and J).
(PDF)

Figure S3 **Analyses of general chemical and physical properties of the key FH19-20 mutant proteins.** **A**, Circular dichroism spectras of the wildtype and mutant FH19-20 proteins were similar indicating that all the tested mutant proteins are most likely folded properly. Crystal structure of the R1203A mutant has been previously published [41] and found to be practically the same as the wildtype FH19-20 structure. **B**, Purified mutant proteins (35 mM) run through a size exclusion gel filtration column appeared in the elute within the same fractions as wildtype FH19-20 implying that the dimerization or oligomerization properties of all the tested mutant proteins were similar to the wildtype.
(PDF)

Figure S4 **Binding of ^125^I-FH19-20 to microbes is enhanced in the presence of C3d.** Binding of radiolabeled FH19-20 to indicated microbes was analyzed in the presence (grey bars) and absence (white bars) of C3d. Data (%) with SD's from a representative experiment performed in triplicates are shown.
(PDF)

Figure S5 **Correlation between FH19-20 binding to microbial proteins OspE, FhbA and Tuf and their enhancing effect on FH-mediated cleavage of the C3b alpha-chain.** Binding of ^125^I-FH19-20 (data from the Figure 4; binding of the wild type FH19-20 to proteins without an inhibitor) is shown as cpm's (±SD) on the x-axis and the amount of C3b alpha chain (data from the cofactor-assays presented in the Figure 6) is shown as percentages (±SD) on the y-axis. OspE binds more FH19-20 than FhbA and Tuf, and enhances most the disappearance of C3b alpha-chain.
(PDF)

Table S1 **Microbial binding sites on FH.** Microbes bind FH using mainly two interaction sites, one in the domains 6–7 and another in the C-terminal domains 19–20 (indicated in blue). Microbial species used in this study are indicated with bold font. The selected references contain information on binding site(s) of FH for each microbe.
(PDF)

## References

[1] Gordon DL, Kaufman RM, Blackmore TK, Kwong J, Lublin DM (1995) Identification of complement regulatory domains in human factor H. J Immunol 155: 348–356. 7541419

[2] Kühn S, Skerka C, Zipfel PF (1995) Mapping of the complement regulatory domains in the human factor H-like protein 1 and in factor H1. J Immunol 155: 5663–5670. 7499851

[3] Weiler JM, Daha MR, Austen KF, Fearon DT (1976) Control of the amplification convertase of complement by the plasma protein beta1H. Proc Natl Acad Sci USA 73: 3268–3272. 1067618 10.1073/pnas.73.9.3268 PMC431003

[4] Whaley K, Ruddy S (1976) Modulation of the alternative complement pathways by beta 1 H globulin. J Exp Med 144: 1147–1163. 62817 10.1084/jem.144.5.1147 PMC2190448

[5] Pangburn MK (2002) Cutting edge: localization of the host recognition functions of complement factor H at the carboxyl-terminal: implications for hemolytic uremic syndrome. J Immunol 169: 4702–4706. 12391176 10.4049/jimmunol.169.9.4702

[6] Jokiranta TS, Cheng ZZ, Seeberger H, Jozsi M, Heinen S, et al (2005) Binding of complement factor H to endothelial cells is mediated by the carboxy-terminal glycosaminoglycan binding site. Am J Pathol 167: 1173–1181. 16192651 10.1016/S0002-9440(10)61205-9 PMC1603661

[7] Jokiranta TS, Jaakola VP, Lehtinen MJ, Pärepalo M, Meri S, et al (2006) Structure of complement factor H carboxyl-terminus reveals molecular basis of atypical haemolytic uremic syndrome. EMBO J 25: 1784–1794. 16601698 10.1038/sj.emboj.7601052 PMC1440827

[8] Kajander T, Lehtinen MJ, Hyvarinen S, Bhattacharjee A, Leung E, et al (2011) Dual interaction of factor H with C3d and glycosaminoglycans in host-nonhost discrimination by complement. Proc Natl Acad Sci USA 108: 2897–2902. 21285368 10.1073/pnas.1017087108 PMC3041134

[9] Jokiranta TS, Zipfel PF, Fremeaux-Bacchi V, Taylor CM, Goodship TJ, et al (2007) Where next with atypical hemolytic uremic syndrome? Mol Immunol 44: 3889–3900. 17768107 10.1016/j.molimm.2007.06.003

[10] Edwards AO, Ritter R 3rd, Abel KJ, Manning A, Panhuysen C, et al (2005) Complement factor H polymorphism and age-related macular degeneration. Science 308: 421–424. 15761121 10.1126/science.1110189

[11] Haines JL, Hauser MA, Schmidt S, Scott WK, Olson LM, et al (2005) Complement factor H variant increases the risk of age-related macular degeneration. Science 308: 419–421. 15761120 10.1126/science.1110359

[12] Lambris JD, Ricklin D, Geisbrecht BV (2008) Complement evasion by human pathogens. Nat Rev Microbiol 6: 132–142. 18197169 10.1038/nrmicro1824 PMC2814840

[13] Blackmore TK, Fischetti VA, Sadlon TA, Ward HM, Gordon DL (1998) M protein of the group A *Streptococcus* binds to the seventh short consensus repeat of human complement factor H. Infect Immun 66: 1427–1431. 9529063 10.1128/iai.66.4.1427-1431.1998 PMC108070

[14] Madico G, Welsch JA, Lewis LA, McNaughton A, Perlman DH, et al (2006) The meningococcal vaccine candidate GNA1870 binds the complement regulatory protein factor H and enhances serum resistance. J Immunol 177: 501–510. 16785547 10.4049/jimmunol.177.1.501 PMC2248442

[15] Zipfel PF, Hellwage J, Friese MA, Hegasy G, Jokiranta ST, et al (1999) Factor H and disease: a complement regulator affects vital body functions. Mol Immunol 36: 241–248. 10403477 10.1016/s0161-5890(99)00038-3

[16] Zipfel PF, Wurzner R, Skerka C (2007) Complement evasion of pathogens: common strategies are shared by diverse organisms. Mol Immunol 44: 3850–3857. 17768102 10.1016/j.molimm.2007.06.149

[17] Kraiczy P, Hellwage J, Skerka C, Becker H, Kirschfink M, et al (2004) Complement resistance of *Borrelia burgdorferi* correlates with the expression of BbCRASP-1, a novel linear plasmid-encoded surface protein that interacts with human factor H and FHL-1 and is unrelated to Erp proteins. J Biol Chem 279: 2421–2429. 14607842 10.1074/jbc.M308343200

[18] Hellwage J, Meri T, Heikkilä T, Alitalo A, Panelius J, et al (2001) The complement regulator factor H binds to the surface protein OspE of *Borrelia burgdorferi* . J Biol Chem 276: 8427–8435. 11113124 10.1074/jbc.M007994200

[19] Hovis KM, McDowell JV, Griffin L, Marconi RT (2004) Identification and characterization of a linear-plasmid-encoded factor H-binding protein (FhbA) of the relapsing fever spirochete *Borrelia hermsii* . J Bacteriol 186: 2612–2618. 15090501 10.1128/JB.186.9.2612-2618.2004 PMC387808

[20] Kunert A, Losse J, Gruszin C, Huhn M, Kaendler K, et al (2007) Immune evasion of the human pathogen *Pseudomonas aeruginosa*: elongation factor Tuf is a factor H and plasminogen binding protein. J Immunol 179: 2979–2988. 17709513 10.4049/jimmunol.179.5.2979

[21] Hallström T, Zipfel PF, Blom AM, Lauer N, Forsgren A, et al (2008) *Haemophilus influenzae* interacts with the human complement inhibitor factor H. J Immunol 181: 537–545. 18566420 10.4049/jimmunol.181.1.537

[22] Amdahl H, Jarva H, Haanpera M, Mertsola J, He Q, et al (2011) Interactions between *Bordetella pertussis* and the complement inhibitor factor H. Mol Immunol 48: 697–705. 21167605 10.1016/j.molimm.2010.11.015

[23] Hammerschmidt S, Agarwal V, Kunert A, Haelbich S, Skerka C, et al (2007) The host immune regulator factor H interacts via two contact sites with the PspC protein of *Streptococcus pneumoniae* and mediates adhesion to host epithelial cells. J Immunol 178: 5848–5858. 17442969 10.4049/jimmunol.178.9.5848

[24] Meri T, Hartmann A, Lenk D, Eck R, Wurzner R, et al (2002) The yeast *Candida albicans* binds complement regulators factor H and FHL- 1. Infect Immun 70: 5185–5192. 12183569 10.1128/IAI.70.9.5185-5192.2002 PMC128257

[25] Hovis KM, Schriefer ME, Bahlani S, Marconi RT (2006) Immunological and molecular analyses of the *Borrelia hermsii* factor H and factor H-like protein 1 binding protein, FhbA: demonstration of its utility as a diagnostic marker and epidemiological tool for tick-borne relapsing fever. Infect Immun 74: 4519–4529. 16861638 10.1128/IAI.00377-06 PMC1539583

[26] Blackmore TK, Hellwage J, Sadlon TA, Higgs N, Zipfel PF, et al (1998) Identification of the second heparin-binding domain in human complement factor H. J Immunol 160: 3342–3348. 9531293

[27] Lehtinen MJ, Rops AL, Isenman DE, van der Vlag J, Jokiranta TS (2009) Mutations of factor H impair regulation of surface-bound C3b by three mechanisms in atypical hemolytic uremic syndrome. J Biol Chem 284: 15650–15658. 19351878 10.1074/jbc.M900814200 PMC2708861

[28] Morgan HP, Schmidt CQ, Guariento M, Blaum BS, Gillespie D, et al (2011) Structural basis for engagement by complement factor H of C3b on a self surface. Nat Struct Mol Biol 18: 463–470. 21317894 10.1038/nsmb.2018 PMC3512577

[29] Janssen BJ, Christodoulidou A, McCarthy A, Lambris JD, Gros P (2006) Structure of C3b reveals conformational changes that underlie complement activity. Nature 444: 213–216. 17051160 10.1038/nature05172

[30] Pangburn MK, Müller-Eberhard HJ (1978) Complement C3 convertase: cell surface restriction of b1H control and generation of restriction on neuraminidase-treated cells. Proc Natl Acad Sci USA 75: 2416–2420. 276881 10.1073/pnas.75.5.2416 PMC392564

[31] Haapasalo K, Vuopio J, Syrjanen J, Suvilehto J, Massinen S, et al (2012) Acquisition of complement factor H is important for pathogenesis of *Streptococcus pyogenes* infections: evidence from bacterial in vitro survival and human genetic association. J Immunol 188: 426–435. 22140259 10.4049/jimmunol.1102545

[32] Hovis KM, Jones JP, Sadlon T, Raval G, Gordon DL, et al (2006) Molecular analyses of the interaction of *Borrelia hermsii* FhbA with the complement regulatory proteins factor H and factor H-like protein 1. Infect Immun 74: 2007–2014. 16552029 10.1128/IAI.74.4.2007-2014.2006 PMC1418896

[33] Meri S, Pangburn MK (1990) Discrimination between activators and nonactivators of the alternative pathway of complement: regulation via a sialic acid/polyanion binding site on factor H. Proc Natl Acad Sci USA 87: 3982–3986. 1692629 10.1073/pnas.87.10.3982 PMC54028

[34] Herbert AP, Deakin JA, Schmidt CQ, Blaum BS, Egan C, et al (2007) Structure shows that a glycosaminoglycan and protein recognition site in factor H is perturbed by age-related macular degeneration-linked single nucleotide polymorphism. J Biol Chem 282: 18960–18968. 17360715 10.1074/jbc.M609636200

[35] Ferreira VP, Herbert AP, Cortes C, McKee KA, Blaum BS, et al (2009) The binding of factor H to a complex of physiological polyanions and C3b on cells is impaired in atypical hemolytic uremic syndrome. J Immunol 182: 7009–7018. 19454698 10.4049/jimmunol.0804031 PMC2696619

[36] Ferreira VP, Pangburn MK, Cortes C (2010) Complement control protein factor H: the good, the bad, and the inadequate. Mol Immunol 47: 2187–2197. 20580090 10.1016/j.molimm.2010.05.007 PMC2921957

[37] Schneider MC, Prosser BE, Caesar JJ, Kugelberg E, Li S, et al (2009) *Neisseria meningitidis* recruits factor H using protein mimicry of host carbohydrates. Nature 458: 890–893. 19225461 10.1038/nature07769 PMC2670278

[38] Kim HK, Thammavongsa V, Schneewind O, Missiakas D (2012) Recurrent infections and immune evasion strategies of *Staphylococcus aureus* . Curr Opin Microbiol 15: 92–99. 22088393 10.1016/j.mib.2011.10.012 PMC3538788

[39] Sjobring U, Bjorck L, Kastern W (1991) Streptococcal protein G. Gene structure and protein binding properties. J Biol Chem 266: 399–405. 1985908

[40] Leo JC, Goldman A (2009) The immunoglobulin-binding Eib proteins from Escherichia coli are receptors for IgG Fc. Mol Immunol 46: 1860–1866. 19303642 10.1016/j.molimm.2009.02.024

[41] Bhattacharjee A, Lehtinen MJ, Kajander T, Goldman A, Jokiranta TS (2010) Both domain 19 and domain 20 of factor H are involved in binding to complement C3b and C3d. Mol Immunol 47: 1686–1691. 20378178 10.1016/j.molimm.2010.03.007

[42] Blanc C, Roumenina LT, Ashraf Y, Hyvärinen S, Sethi SK, et al (2012) Overall Neutralization of Complement Factor H by Autoantibodies in the Acute Phase of the Autoimmune Form of Atypical Hemolytic Uremic Syndrome. J Immunol 189 (7) 3528–3. 22922817 10.4049/jimmunol.1200679

[43] Koistinen V, Wessberg S, Leikola J (1989) Common binding region of complement factors B, H and CR1 on C3b revealed by monoclonal anti-C3d. Complement Inflamm 6: 270–280. 2527715 10.1159/000463102

[44] Wu J, Wu YQ, Ricklin D, Janssen BJ, Lambris JD, et al (2009) Structure of complement fragment C3b-factor H and implications for host protection by complement regulators. Nat Immunol 10: 728–733. 19503104 10.1038/ni.1755 PMC2713992

