# Correction: Microbes Bind Complement Inhibitor Factor H *via* a Common Site

**DOI:** 10.1371/annotation/41169409-3260-4295-baf4-a1a4621a8e48

**Published:** 2013-04-24

**Authors:** T. Meri, H. Amdahl, M. J. Lehtinen, S. Hyvärinen, J. V. McDowell, A. Bhattacharjee, S. Meri, R. Marconi, A. Goldman, T. S. Jokiranta

The affiliation for author T. S. Jokiranta is incorrect. T. S. Jokiranta's affiliation should instead be: Haartman Institute, Department of Bacteriology and Immunology and Immunobiology Research Program, University of Helsinki, Helsinki, Finland

