# Supplementary figures and images for: Microbes Bind Complement Inhibitor Factor H via a Common Site
Source: PLoS Pathog. 2013 Apr 18;9(4):e1003308. doi: 10.1371/journal.ppat.1003308 (PMC3630169; doi:10.1371/journal.ppat.1003308)

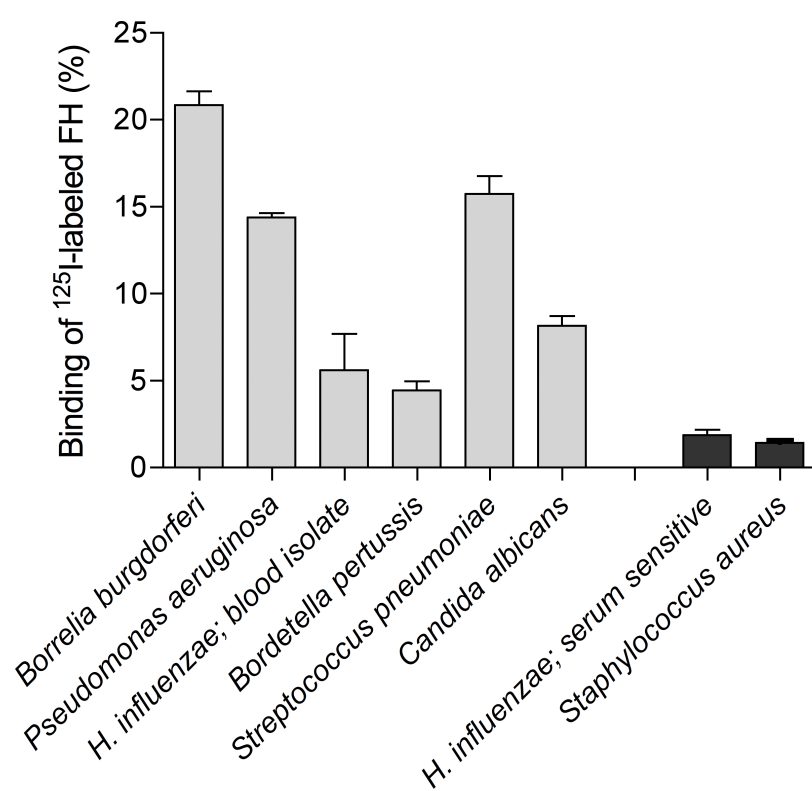

Supplement: Figure S1 — Binding of full length FH to microbes used in the study. Bacteria and yeast (1×10^8^/assay) were incubated with radiolabeled FH and samples were centrifuged through sucrose colums to separate unbound radioactivity. Amount of radioactivity in the pellet and supernatant was measured with a gamma-counter and FH bound to the microbes is shown as a percentage from total amount of protein given. Data (%) with SD's from a representative experiment performed in triplicates are shown. As negative controls a serum sensitive strain of H. influenzae and. S. aureus were used. (PDF) [file ppat.1003308.s001.pdf]

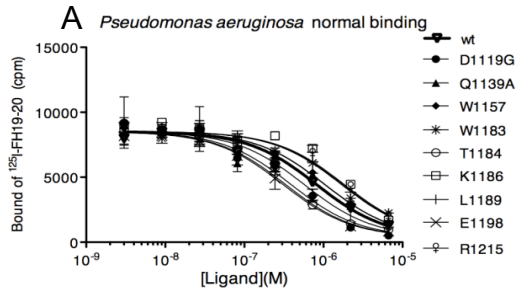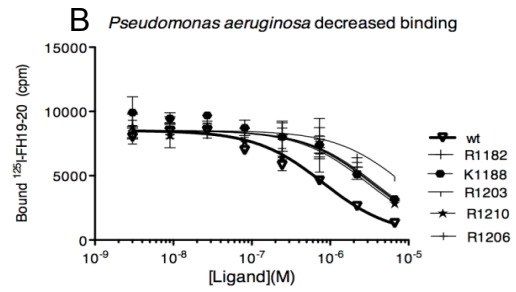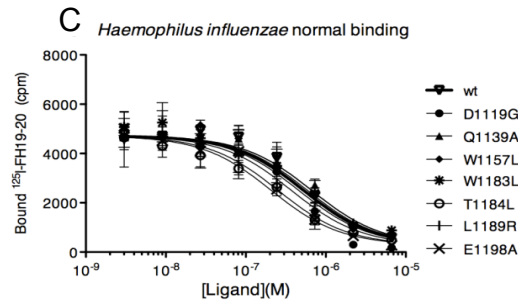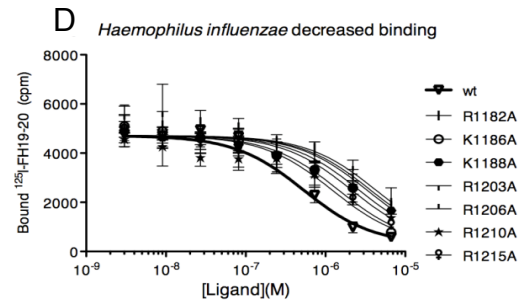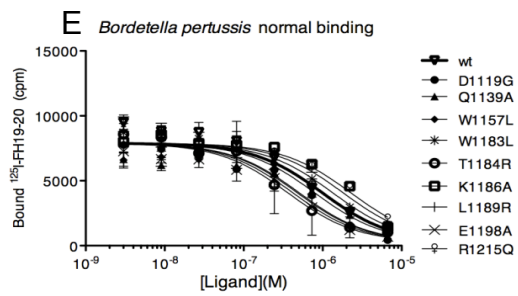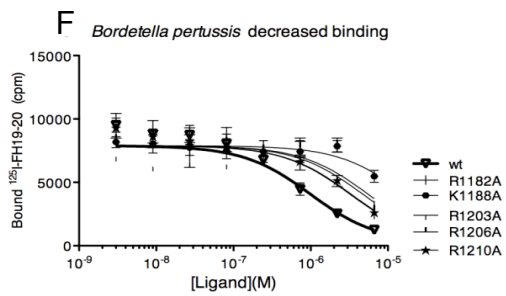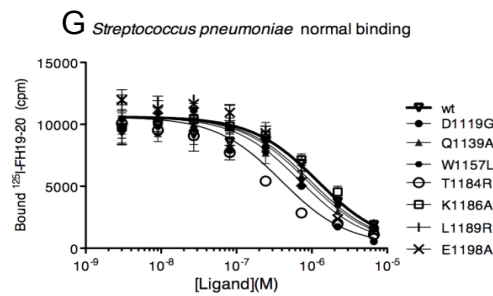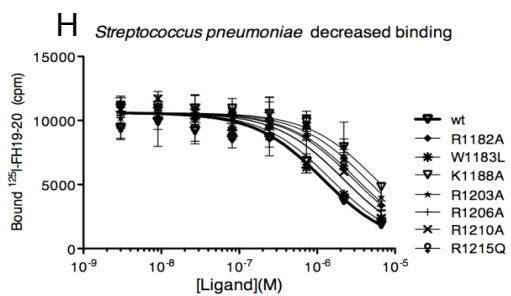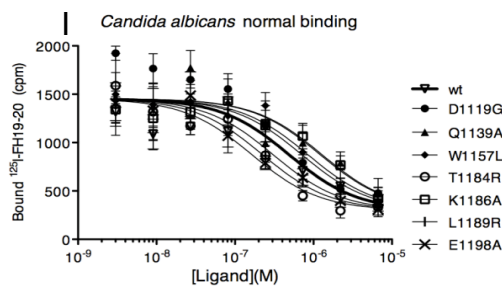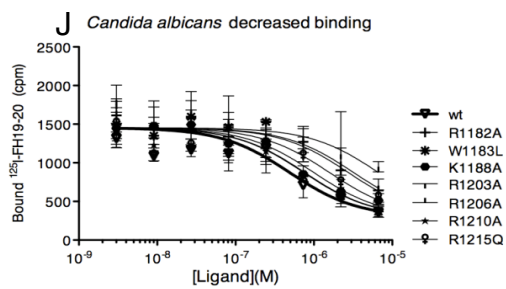

Supplement: Figure S2 — Examples of the inhibition assays. Curves from a single out of three experiments (performed in triplicates) where inhibition of 125FH19-20 binding to various microbes by wildtype (wt) and mutant FH19-20 proteins was analyzed to obtain IC50 values (shown in Figure 1 ). The used microbes were Pseudomonas aeruginosa (panels A and B), Haemophilus influenzae (panels C and D), Bordetella pertussis (panels E and F), Streptococcus pneumoniae (panels G and H), and Candida albicans (panels I and J). (PDF) [file ppat.1003308.s002.pdf]

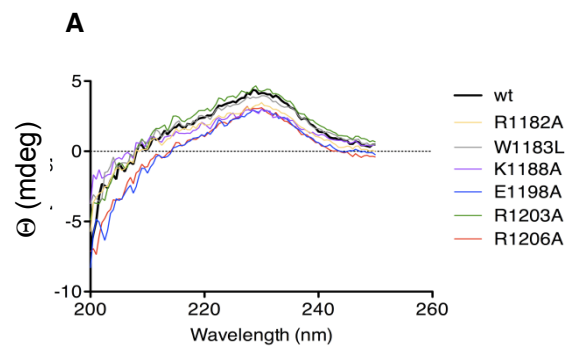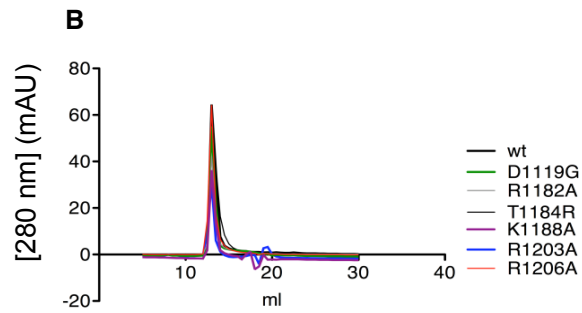

Supplement: Figure S3 — Analyses of general chemical and physical properties of the key FH19-20 mutant proteins. A, Circular dichroism spectras of the wildtype and mutant FH19-20 proteins were similar indicating that all the tested mutant proteins are most likely folded properly. Crystal structure of the R1203A mutant has been previously published [41] and found to be practically the same as the wildtype FH19-20 structure. B, Purified mutant proteins (35 mM) run through a size exclusion gel filtration column appeared in the elute within the same fractions as wildtype FH19-20 implying that the dimerization or oligomerization properties of all the tested mutant proteins were similar to the wildtype. (PDF) [file ppat.1003308.s003.pdf]

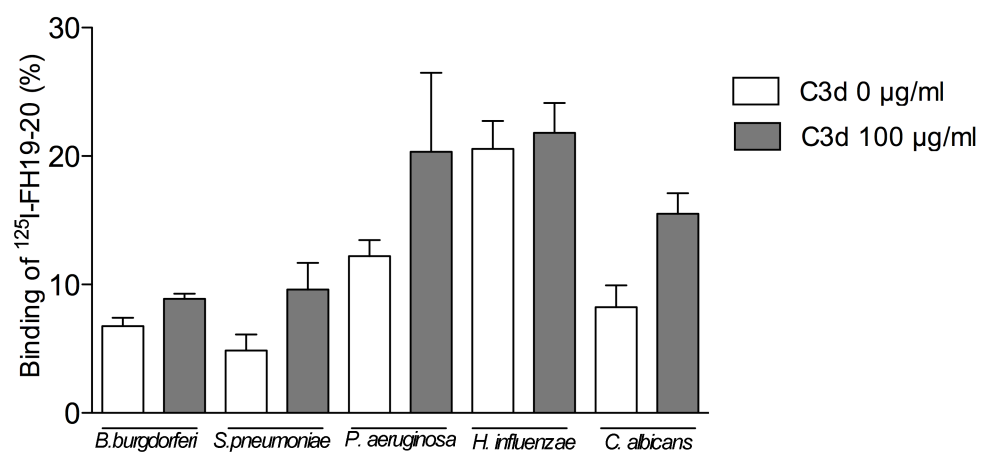

Supplement: Figure S4 — Binding of 125I-FH19-20 to microbes is enhanced in the presence of C3d. Binding of radiolabeled FH19-20 to indicated microbes was analyzed in the presence (grey bars) and absence (white bars) of C3d. Data (%) with SD's from a representative experiment performed in triplicates are shown. (PDF) [file ppat.1003308.s004.pdf]

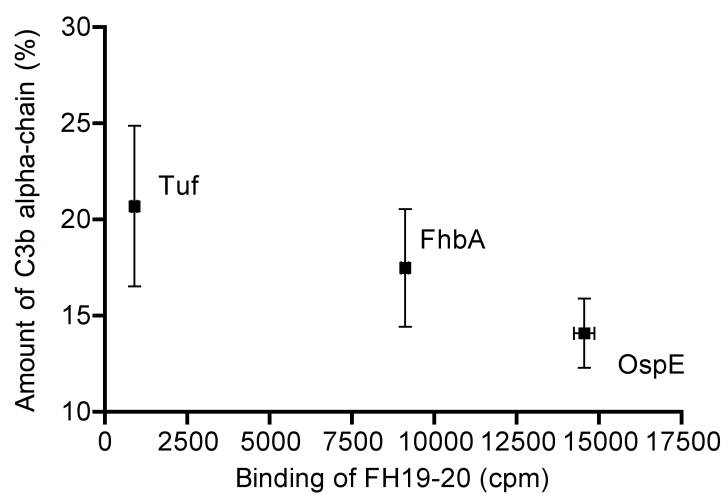

Supplement: Figure S5 — Correlation between FH19-20 binding to microbial proteins OspE, FhbA and Tuf and their enhancing effect on FH-mediated cleavage of the C3b alpha-chain. Binding of 125I-FH19-20 (data from the Figure 4; binding of the wild type FH19-20 to proteins without an inhibitor) is shown as cpm's (±SD) on the x-axis and the amount of C3b alpha chain (data from the cofactor-assays presented in the Figure 6) is shown as percentages (±SD) on the y-axis. OspE binds more FH19-20 than FhbA and Tuf, and enhances most the disappearance of C3b alpha-chain. (PDF) [file ppat.1003308.s005.pdf]
