# Supplementary material for: Microbes Bind Complement Inhibitor Factor H via a Common Site
Source: PLoS Pathog. 2013 Apr 18;9(4):e1003308. doi: 10.1371/journal.ppat.1003308 (PMC3630169; doi:10.1371/journal.ppat.1003308)
Supplement: Table S1 — Microbial binding sites on FH. Microbes bind FH using mainly two interaction sites, one in the domains 6–7 and another in the C-terminal domains 19–20 (indicated in blue). Microbial species used in this study are indicated with bold font. The selected references contain information on binding site(s) of FH for each microbe. (PDF) [file ppat.1003308.s006.pdf]

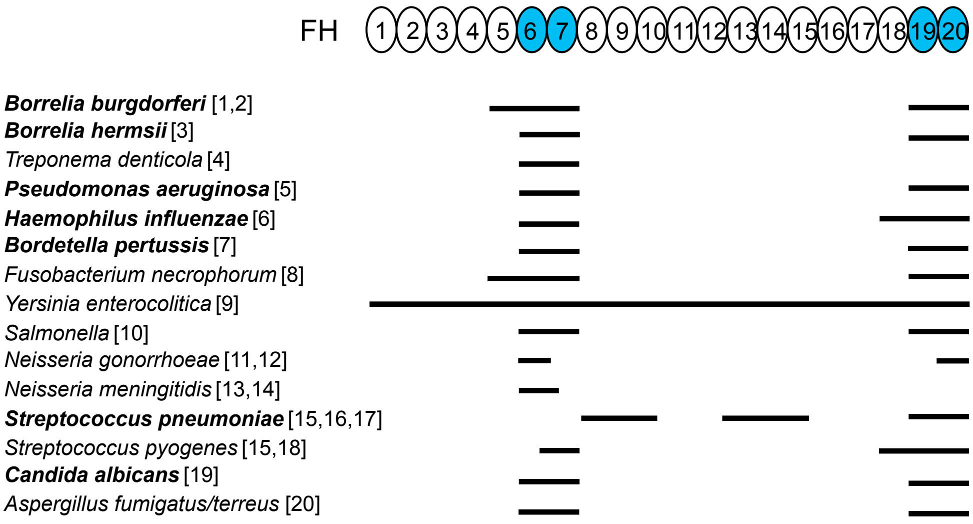

1. Kraiczy P, Hellwage J, Skerka C, Kirschfink M, Brade V, et al. (2003) Immune evasion of *Borrelia burgdorferi*: mapping of a complement-inhibitor factor H-binding site of BbCRASP-3, a novel member of the Erp protein family. Eur J Immunol 33: 697-707.
2. Alitalo A, Meri T, Chen T, Lankinen H, Cheng ZZ, et al. (2004) Lysine-dependent multipoint binding of the *Borrelia burgdorferi* virulence factor outer surface protein E to the C terminus of factor H. J Immunol 172: 6195-6201.
3. Hovis KM, McDowell JV, Griffin L, Marconi RT (2004) Identification and characterization of a linear-plasmid-encoded factor H-binding protein (FhbA) of the relapsing fever spirochete *Borrelia hermsii*. J Bacteriol 186: 2612-2618.
4. McDowell JV, Lankford J, Stamm L, Sadlon T, Gordon DL, et al. (2005) Demonstration of factor H-like protein 1 binding to *Treponema denticola*, a pathogen associated with periodontal disease in humans. Infect Immun 73: 7126-7132.
5. Kunert A, Losse J, Gruszin C, Huhn M, Kaendler K, et al. (2007) Immune evasion of the human pathogen *Pseudomonas aeruginosa*: elongation factor Tuf is a factor H and plasminogen binding protein. J Immunol 179: 2979-2988.
6. Hallstrom T, Zipfel PF, Blom AM, Lauer N, Forsgren A, et al. (2008) *Haemophilus influenzae* interacts with the human complement inhibitor factor H. J Immunol 181: 537-545.
7. Amdahl H, Jarva H, Haanperä M, Mertsola J, He Q, et al. (2011) Interactions between *Bordetella pertussis* and the complement inhibitor factor H. Mol Immunol 48: 697-705.
8. Friberg N, Carlson P, Kentala E, Mattila PS, Kuusela P, et al. (2008) Factor H binding as a complement evasion mechanism for an anaerobic pathogen, *Fusobacterium necrophorum*. J Immunol 181: 8624-8632.
9. Biedzka-Sarek M, Salmenlinna S, Gruber M, Lupas AN, Meri S, et al. (2008) Functional mapping of YadA- and Ail-mediated binding of human factor H to *Yersinia enterocolitica* serotype O:3. Infect Immun 76: 5016-5027.
10. Ho DK, Jarva H, Meri S Human complement factor H binds to outer membrane protein Rck of Salmonella. J Immunol 185: 1763-1769.
11. Ram S, McQuillen DP, Gulati S, Elkins C, Pangburn MK, et al. (1998) Binding of complement factor H to loop 5 of porin protein 1A: a molecular mechanism of serum resistance of nonsialylated *Neisseria gonorrhoeae*. J Exp Med 188: 671-680.
12. Ngampasutadol J, Ram S, Gulati S, Agarwal S, Li C, et al. (2008) Human factor H interacts selectively with *Neisseria gonorrhoeae* and results in species-specific complement evasion. J Immunol 180: 3426-3435.
13. Lewis LA, Ngampasutadol J, Wallace R, Reid JE, Vogel U, et al. (2010) The meningococcal vaccine candidate neisserial surface protein A (NspA) binds to factor H and enhances meningococcal resistance to complement. PLoS Pathog 6: e1001027.
14. Schneider MC, Prosser BE, Caesar JJ, Kugelberg E, Li S, et al. (2009) *Neisseria meningitidis* recruits factor H using protein mimicry of host carbohydrates. Nature 458: 890-893.
15. Blackmore TK, Fischetti VA, Sadlon TA, Ward HM, Gordon DL (1998) M protein of the group A Streptococcus binds to the seventh short consensus repeat of human complement factor H. Infect Immun 66: 1427-1431.
16. Jarva H, Hellwage J, Jokiranta TS, Lehtinen MJ, Zipfel PF, et al. (2004) The group B streptococcal beta and pneumococcal Hic proteins are structurally related immune evasion molecules that bind the complement inhibitor factor H in an analogous fashion. J Immunol 172: 3111-3118.
17. Hammerschmidt S, Agarwal V, Kunert A, Haelbich S, Skerka C, et al. (2007) The host immune regulator factor H interacts via two contact sites with the PspC protein of *Streptococcus pneumoniae* and mediates adhesion to host epithelial cells. J Immunol 178: 5848-5858.
18. Reuter M, Caswell CC, Lukomski S, Zipfel PF (2010) Binding of the human complement regulators CFHR1 and factor H by streptococcal collagen-like protein 1 (Scl1) via their conserved C termini allows control of the complement cascade at multiple levels. J Biol Chem 285: 38473-38485.
19. Meri T, Hartmann A, Lenk D, Eck R, Wurzner R, et al. (2002) The yeast *Candida albicans* binds complement regulators factor H and FHL- 1. Infect Immun 70: 5185-5192.
20. Behnsen J, Hartmann A, Schmaler J, Gehrke A, Brakhage AA, et al. (2008) The opportunistic human pathogenic fungus *Aspergillus fumigatus* evades the host complement system. Infect Immun 76: 820-827.
